# Supplementary material for: Psilocybin-assisted therapy for the treatment of resistant major depressive disorder (PsiDeR): protocol for a randomised, placebo-controlled feasibility trial
Source: BMJ Open. 2021 Dec 1;11(12):e056091. doi: 10.1136/bmjopen-2021-056091 (PMC8638462; doi:10.1136/bmjopen-2021-056091)
Supplement: Supplementary data [file bmjopen-2021-056091supp001.pdf]

Supplementary Information for:

Psilocybin-assisted therapy for the treatment of resistant major depressive disorder (PsiDeR): protocol for a randomised, placebo-controlled feasibility trial

1. Authors

James J Rucker[1,2], Hassan Jafari[3], Tim Mantingh[1], Catherine Bird[1], Nadav Liam Modlin[1], Gemma Knight[1], Frederick Reinholdt[1], Camilla Day[1,2], Ben Carter[3], Allan H Young[1,2].

2. Affiliations

1. The Department of Psychological Medicine, The Institute of Psychiatry, Psychology & Neuroscience, King's College London, 16 De Crespigny Park, London, SE5 8AF, UK
2. The South London & Maudsley NHS Foundation Trust, Denmark Hill, London, SE5 8AZ, UK
3. Department of Biostatistics and Health Informatics, The Institute of Psychiatry, Psychology & Neuroscience, King's College London, 16 De Crespigny Park, London, SE5 8AF

3. Trial Objectives

3.1. Primary Objective

To evaluate the feasibility of a randomised, controlled trial design, in which a single dose of psilocybin 25mg PO vs placebo, is given to adult participants with treatment resistant major depressive disorder (TRD), under psychologically supportive conditions, with 6 weeks of follow up, by measuring recruitment rates, dropout rates and by estimating the variance of the primary outcome measure (MADRS) to inform upon the design of a phase 3 trial.

3.2. Secondary Objectives

The secondary objectives of this trial are as follows:

1) To assess the clinician-rated efficacy of psilocybin 25mg compared to placebo via:
   a) The change in the Montgomery Asberg Depression Rating Scale (MADRS) total score from Baseline to 3 weeks after treatment.
   b) The proportion of participants who demonstrate a response to treatment, where response to treatment is defined as a ≥50% decrease in MADRS total score from Baseline to Week 3. This will also be assessed at Weeks 1 and 6.
   c) The proportion of participants in remission, where remission is defined as a MADRS total score ≤10 at Week 3. This will also be assessed at Weeks 1 and 6.
2) To assess the participant-rated efficacy of psilocybin 25mg compared to placebo via:
   a) The proportion of participants who demonstrate a response to treatment, where response to treatment is defined as a ≥50% decrease in QIDS-SR-16 total score from Baseline (V2) to Week 3 (V6).
   b) The proportion of participants in remission, where remission is defined as a QIDS-SR-16 total score ≤5 at Week 3. This will also be assessed at Weeks 1 and 6.
3) To evaluate the safety and tolerability of psilocybin in participants with TRD based on adverse events (AEs), changes in vital signs and suicidal ideation/behaviour (measured using the Columbia Suicide Severity Rating Scale).

3.3. Exploratory Objectives

1) Explore the acceptability, tolerability and safety of delivering >1 dose of psilocybin by collecting safety data and participant narrative data from an optional 12 week open label extension to the trial.

4. Trial Endpoints

4.1. Primary Endpoint

The primary endpoint in this study will be:

Recruitment rates, dropout rates and estimation of the variance of the primary outcome measure (MADRS).

4.2. Secondary Endpoints

The secondary endpoints in this study will be:

1) The change in the Montgomery-Asberg Depression Rating Scale total score from the Baseline Visit (V2 - 1 day prior to treatment) to Week 3 after treatment (V6).
2) The proportion of participants who demonstrate a response to treatment, where response to treatment is defined as a ≥50% decrease in MADRS total score from Baseline to Week 3.
3) The proportion of participants in remission, where remission is defined as a MADRS total score ≤10 at Week 3.
4) The proportion of participants who sustain a response up to Week 6, where response is defined as those with ≥50% decrease in MADRS total score on or before Week 3 and remaining at Week 6.
5) Time to event measures: restart antidepressant medication for any reason, restart medication for continuing depressive symptoms or relapse from a previously recovered state (clinical judgement, supported by the QIDS-SR-16). Participants who withdraw from the study will be censored from the time to event analysis.

4.3. Exploratory Endpoints

This study will include the following exploratory endpoints:

1) Change from baseline (V2) in the following outcome measures
   a) The WSAS at week 6 (V7)
   b) The QIDS-SR-16 at week 3 (V6)
   c) The GAD-7 at week 3 (V6)

5. Trial Procedures

5.1. Trial Measures & Procedures

Trial measures and procedures are listed in Table 4, whilst a schedule of activities is listed in Table 5. Note that the times listed in Table 5 denote the estimated time taken for participant completion AND the estimated time taken for the study team to administer subsequently (data input, cleaning etc.).

5.1.1. List of Measures & Procedures

Measure/Procedure	Type	Reference	Primary Rationale	Administration Schedule
Informed Consent	Study Team-administered	Protocol	Screening	V1, [OLE1]
Medical History	Study Team-administered	Protocol	Screening	V1, V2, V3
Inclusion/Exclusion Criteria	Study Team-administered	Protocol	Screening	V1, V2, [OLE1]
Prior & Concomitant Medications	Study Team-administered	Protocol	Safety	V1, V2, V3, V4, V5, V6, V7, [OLE1, OLE2, OLE3, OLE4, OLE5, OLE6, OLE7]
Physical Examination	Study Team-administered	Protocol	Safety	V1, (V2, V3, V4, V5, V6, V7, [OLE1, OLE2, OLE3, OLE4, OLE5, OLE6, OLE7] if indicated)
Collateral History	Study Team-administered	Protocol	Safety	V1, V2, [OLE1]
Adverse Events (UKU/HPPDS)	Study Team-administered	Protocol & [1]	Safety	V1, V2, V3, V4, V5, V6, V7, [OLE2, OLE3, OLE4, OLE5, OLE6, OLE7]
Clinical laboratory investigations	Study Team-administered	Protocol	Screening	V1, V2, V4, [OLE3]
Electrocardiogram	Study Team-administered	Protocol	Screening	V1, V4, [OLE3]
Vital signs (BP/HR/Temp)	Study Team-administered	Protocol	Safety	V1, V2, V3 V4, [OLE1, OLE2, OLE3]
Weight	Study Team-administered	Protocol	Safety	V1, V2, V7
Urinalysis	Study Team-administered	Protocol	Screening	V1
Urine Drug Screen	Study Team-administered	Protocol	Screening	V1, [OLE1]
Serum/Urine Pregnancy Test (females only)	Study Team-administered	Protocol	Safety	V1, V2, [OLE1]
Additional biological samples (optional)	Study Team-administered	Protocol	Mechanism	V2, V4, V5, V6, V7
Neuroimaging (optional)	Study Team-administered	Protocol	Mechanism	V2, V5
Fitness watch/phone data	Study Team-administered	Protocol	Outcome	V7, [OLE 6]
Psychological Support & SI	Study Team-administered	Protocol	Support	V2, V3, V4, V5, V6, V7, [OLE1, OLE2, OLE3, OLE4, OLE5, OLE6, OLE7]
Treatment	Study Team-administered	Protocol	Intervention	V2, [OLE2]
MINI	Study Team-administered	[2]	Screening	V1
HAM-D-17	Study Team-administered	[3]	Screening	V1, V2
Maudsley Staging Method	Study Team-administered	[4]	Screening	V1
MADRS	Study Team-administered	[5]	Outcome	V2, V5, V6, V7, [OLE4, OLE5, OLE6, OLE7]
CSSRS	Study Team-administered	[6]	Safety	V1, V2, V4, V5, V6, V7, [OLE3, OLE4, OLE5, OLE6, OLE7]

YMRS	Study Team-administered	[7]	Safety	V2, V4, V5, V6, V7, [OLE3, OLE4, OLE5, OLE6, OLE7]
STAR-C	Study Team-administered	[8]	Mechanism	V2, V5, V6, V7, [OLE4, OLE5, OLE6]
QIDS-SR-16	Participant-administered	[9]	Outcome	V1, V2, V5, V6, V7, [OLE4, OLE5, OLE6, OLE7]
GAD-7	Participant-administered	[10]	Outcome	V2, V5, V6, V7, [OLE4, OLE5, OLE6, OLE7]
CTS	Participant-administered	[11]	Mechanism	V2
STAI	Participant-administered	[12]	Mechanism	V2, V5, V6, V7, [OLE5, OLE6, OLE7]
STAR-P	Participant-administered	[8]	Mechanism	V2, V5, V6, V7, [OLE5, OLE6, OLE7]
DESS	Participant-administered	[13]	Safety	V2
5D-ASC	Participant-administered	[14]	Mechanism	V4, [OLE3]
ASRM	Participant-administered	[15]	Safety	V2, V5, V6, V7, [OLE4, OLE5, OLE6, OLE7]
SCL-90	Participant-administered	[16]	Safety	V2, V5
DePAS	Participant-administered	Protocol	Safety	V5, V6, V7, [OLE5, OLE6, OLE7]
EQ-5D-5L	Participant-administered	[17]	Outcome	V2, V6, V7, [OLE5, OLE6, OLE7]
WSAS	Participant-administered	[18]	Outcome	V1, V2, V6, V7, [OLE5, OLE6, OLE7]
TAS	Participant-administered	[19]	Mechanism	V2, V7, [OLE6]
PANAS	Participant-administered	[20]	Safety	V2, V4, V5, V6, V7, [OLE3, OLE4, OLE5, OLE6, OLE7]
SCSS	Participant-administered	[21]	Mechanism	V2, V4, V7, [OLE3]
SCST	Participant-administered	Protocol	Mechanism	V2, V6, V7, [OLE5, OLE6]
CES	Participant-administered	Protocol	Mechanism	V4. [OLE3]
TSDI	Participant-administered	Protocol	Mechanism	V1, V2, V7
WEMWBS	Participant-administered	[22]	Mechanism	V2, V6, V7, [OLE5, OLE6]
CNS	Participant-administered	[23]	Mechanism	V2, V6, V7, [OLE5, OLE6]
SAPAS	Participant-administered	[24]	Mechanism	V1, V2, V7, [OLE6]
MTI	Participant-administered	[25]	Screening	V1
THINC-it	Participant-administered	[26]	Mechanism	V1, V2, V5, V7, [OLE4], [OLE6]
ETB	Participant-administered	[27]	Mechanism	V2, V5, V7, [OLE4, OLE6]
MVAS	Participant-administered	Protocol	Mechanism	V1, V2, V4, V5, V6, V7, [OLE3, OLE4, OLE5, OLE6]

Table 1. List of study related procedures and measures. [X] Refers to procedures that will only be undertaken if the participant gives informed consent to the Open Label Extension part of the protocol.

5.1.2. Schedule of Measures & Procedures

Study Team Administered Measures and Procedures													
**Visit**	**V1**	**V2**	**V3**	**V4**	**V5**	**V6**	**V7/ OLE1**	**OLE2**	**OLE3**	**OLE4**	**OLE5**	**OLE6**	**OLE 7**
**Day**	**-56 - -7**	**-1**	**0**	**1**	**7**	**21**	**41**	**42#**	**43**	**49**	**63**	**84**	**126**
**Week**	**-8 - -1**	**0**	**0**	**0**	**1**	**3**	**6**	**6#**	**6**	**7**	**9**	**12**	**18**
**Location**	**KCH Clinical Research Facility**												
**Window**	**N/A**	**-3**	**N/A**	**+1**	**+/- 1**	**+/- 2**	**+7/- 3**	**NA**	**+/- 1 ****	**+/- 1 ****	**+/- 2 ****	**+/- 3 ****	**+/- 7 ****
**Length of Visit (hrs)***	**4**	**6**	**8**	**4**	**6**	**4**	**4**	**8**	**4**	**2**	**4**	**2**	**2 r**
Informed Consent	X						(X)						
Medical History	X	X	X										
Inc/Exc criteria	X	X					(X)						
Prior/concom. meds	X	X	X	X	X	X	X	X	X	X	X	X	X
Physical examination	X	X ***	X ***	X ***	X ***	X ***	X ***	X ***	X ***	X ***	X ***	X ***	X ***
Collateral	X	X					X						
AE/SAE	X	X	X	X	X	X	X	X	X	X	X	X	X
Clinical lab. blood tests	X	X		X					X				
ECG	X			X					X				
Vitals	X	X	X	X			(X)	X	X				
Weight	X	X					X					X	
Urinalysis	X												
Urine drug screen	X						(X)						
Urine/serum pregnancy test	X	X					(X)						
Bloods (additional)		X		X	X	X	X						
Neuroimaging		X			X								
Mobile phone/fitness watch							X					X	
Psych. support & SI (flexible schedule +/- remote)		X	X	X	X	X	X	X	X	X	X	X	X
IMP			X					X					
MINI	X												
HAM-D-17	X	X											
MSM	X												
MADRS		X			X	X	X			X	X	X	X
CSSRS	X	X		X	X	X	X		X	X	X	X	X
YMRS		X		X	X	X	X		X	X	X	X	
MTI*	X												
STAR-C		X			X	X	X			X	X	X	
**Participant Administered Measures and Procedures**													
QIDS-SR-16	X	X			X	X	X			X	X	X	X
GAD-7		X			X	X	X			X	X	X	X
CTS		X											
STAI		X			X	X	X			X	X	X	
STAR-P		X			X	X	X			X	X	X	
DESS		X											
XD-ASC				X					X				
ASRM		X			X	X	X			X	X	X	X
SCL-X		X			X								
DePAS					X	X	X			X	X	X	
EQ-XD-XL		X				X	X				X	X	X
WSAS	X	X				X	X				X	X	X
TAS		X					X					X	
PANAS		X		X	X	X	X		X	X	X	X	X
SCSS		X		X			X		X				

SCST		X				X	X				X	X	
CES				X					X				
TSDI		X					X						
WEMWBS		X				X	X				X	X	
CNS		X				X	X				X	X	
SAPAS	X	X					X					X	
THINC-it	X	X			X		X			X		X	
ETB		X			X		X			X		X	
MVAS	X	X		X	X	X	X		X	X	X	X	
(X) = If consent to OLE given	r = remote visit available				SI = Structured Interview				*joint completion with clinician				
	#up to 5 weeks after V7, if logistically necessary				***if clinically indicated				**relative to date of OLE2				
Medic Task	Nurse/RA Task				Therapist Task				Participant Task				

Table 2. List of Study-Team and Participant-Administered Activities and Measures.

All study visits will take place at the NIHR/Wellcome Trust Clinical Research Facility at King's College Hospital, Denmark Hill, London, UK.

5.1.3. Allowable Windows

Allowable windows for study visits are stated in Table 5, which are implemented to allow a degree of logistical flexibility for participants who may have other commitments and for the study team, who will be sharing facilities with other trials. This reflects 'real-world' clinical conditions and may improve retention and adherence to study protocols. The long allowable window at V7 is to allow for scheduled public holidays over which the university and trial facility is closed (Christmas and Easter) to be accommodated prior to the baseline visit for the open label extension.

5.1.4. Description of Study Team Administered Measures

5.1.4.1. Informed Consent

Informed consent will be gained by a member of the study team at V1. Participants will be provided with a participant information leaflet to read prior to informed consent being sought. As much time as necessary will be given to address any questions or concerns from participants. Final eligibility and consent will be determined by a clinician.

5.1.4.2. Medical History

A medical history will be taken by a member of the study team at V1 in liaison with a clinician if necessary and reconfirmed at V2 and V3, informed by collateral information gained from the participant's general practitioner or secondary care team.

5.1.4.3. Inclusion/Exclusion Criteria

Inclusion and exclusion criteria will be determined by a member of the study team at V1 and informed by collateral information gained from the participant's general practitioner or secondary care team during the screening and preparation phase. Final confirmation will occur at V2.

5.1.4.4. Prior & Concomitant Medications

Prior and concomitant medications will be determined by a member of the study team in liaison with a clinician if necessary and informed by collateral information gained from the participant's general practitioner or secondary care team. This information will be gained at V1 and then updated at all subsequent visits.

5.1.4.5. Physical Examination

A physical examination must be conducted at screening to confirm participant eligibility and at any other moment if clinically indicated.

5.1.4.6. Adverse Events (UKU/HPPDS)

Adverse events will be determined by a member of the study team in liaison with a clinician if necessary. Adverse events will be collected from consent. Adverse events occurring at V1 will be recorded in the medical history. Adverse events occurring after V1 but before dosing will be recorded as adverse events but the causality will be documented as '1' (not related). We will use open questions to enquire how participants have been since last seen by the study team and then document adverse events. Subsequent to dosing, we will use a modified version of the UKU schedule of side effects to psychotropic drugs to standardise closed questions of AE data [1] with an expansion on neuropsychiatric side effects as specified by the following items: 1.11 General anxiety, 1.12 Panic attacks, 1.13 Derealisation, 1.14 Depersonalisation, 1.15 Flashbacks, 1.16 Obsessions, 1.17 Compulsions, 1.18 Elation, 1.19 Irritability, 1.20 Aggression, 1.21 Paranoia / suspiciousness, 1.22 Thought disorder, 1.23 Delusions, 1.24 Visual disturbances, 1.25 Visual hallucinations, 1.26 Auditory disturbances, 1.27 Auditory hallucinations, 1.28 Gustatory disturbances, 1.29 Gustatory hallucinations, 1.30 Olfactory disturbances, 1.31 Olfactory hallucinations, 1.32 Somatic disturbances, 1.33 Somatic hallucinations, 1.34 Loss of insight. Symptoms of hallucinogen persisting perception disorder (HPPD) will also be probed using an operationalised checklist of HPPD criteria derived from DSM5. Adverse event data will be collected at all visits, except that the UKU will be collected only once at V5 to ease participant burden and the HPPDS will not be collected at V4.

5.1.4.7. Clinical Laboratory Investigations

Routine clinical laboratory investigations will be taken at V1, V2 and V4. Bloods will be processed at KHP's onsite laboratories. Parameters evaluated will be: Haematology (Haemoglobin, Red blood cell count, Platelet count, white blood cell count and differential count), Clinical chemistry (Sodium, Potassium, Creatinine, Urea); Liver function test (Total bilirubin, Alanine Aminotransferase, Aspartate Aminotransferase, Alkaline phosphatase); T3, T4 and thyroid stimulating hormone. Results will be reviewed by a clinician.

5.1.4.8. Electrocardiogram

An electrocardiogram will be taken at V1 & V4. ECGs will be reviewed by a clinician.

5.1.4.9. Vital Signs (RR/SaO2/BP/HR/Temp)

Vital signs (respiratory rate, blood oxygen saturations on air, blood pressure, heart rate and temperature) will be taken at V1, V2, V3 & V4. Abnormal readings will be reviewed by a clinician in line with KHP policy.

5.1.4.10. Weight

Weight will be taken at V1, V2 and V7.

5.1.4.11. Urinalysis

Urinalysis will be taken at V1. Abnormal findings will be reviewed by a clinician.

5.1.4.12. Urine Drug Screen

A urine drug screen will be taken at V1. Positive findings will be reviewed by a clinician.

5.1.4.13. Serum/Urine Pregnancy Test (females only)

A serum pregnancy test will be taken at screening and a urine pregnancy test at V2 for pre-menopausal females only. For those pre-menopausal female participants choosing to take part in the open label extension, an additional urine pregnancy test will be undertaken at V7. Positive tests will be reviewed by a clinician and women confirmed to be pregnant will be withdrawn from the study.

5.1.4.14. Additional Bloods (optional)

An optional blood sample for processing and storage will be taken at V2, V4, V5, V6 and V7. Up to 50 mls of whole blood will be drawn.

5.1.4.15. Neuroimaging (optional)

Optional neuroimaging scans (MRI) will be offered at V2 and V5. Abnormal findings will be reviewed by a clinician. Each scan will last 1 hour.

5.1.4.16. Fitness Watch/Mobile Activity Data

If participants already have a fitness watch, or if they are collecting data via their mobile phone, or another device, then we will ask for their consent to share step counts per hour per day and sleep data (time in bed, time in light sleep, time in deep sleep) with us. They will be under no obligation to do so. The analysis of this data is not an intended outcome of this study. Data will only be collected from time points subsequent to the participant signing the ICF.

5.1.4.17. Psychological Support

Psychological support will be offered at V2, V3, V4, V5, V6 & V7. Additional psychological support will be available to participants on request. An element of flexibility in the schedule and provision of psychological support is necessary in a vulnerable population and trial such as this. As far as possible, participants will be supported by the same therapist throughout the trial. Participants will be able to access their therapist via a secure remote video or audio link if a face to face visit is not possible at the actual study visit, or if it is preferred by the participant. Psychological support is

provided by the same therapist throughout a participant's journey through the trial, to the extent that this is possible.

The psychological model used in this trial will be similar to a broader model that has been developed and authorised separately for the Compass Pathways multicentre RCTs in depression. The centre delivering this trial is also a training centre for the delivery of therapists for the Compass multicentre RCTs (as well as being a centre for the trial itself), therefore the therapist trainers in this trial have been trained as part of our existing trials and have unique and experience of working with this patient group in this setting. Training consists of didactic teaching, role play and, if feasible, practical experience in a clinical trial setting supervised by senior psychological therapists with extensive experience of delivering psilocybin therapy within trials performed both here at King's College London and at Johns Hopkins University Hospital in Maryland, USA. The therapist's training manual was reviewed and authorised separately as part of the NREC and MHRA's review of Compass Pathways' multicentre TRD trial with psilocybin.

The psychological model is divided into three phases that reflect preparation, dosing and integration respectively.

Preparation focusses on building mutual trust, active listening, psychoeducation about the drug experience and teaching participants methods of coping with difficult or unusual experiences that might arise. We will require participants to undergo a minimum of three hours of preparation, which may be delivered over a number of separate sessions. This will be assessed prior to dosing and the amount of preparation recorded in the case record form.

Dosing focusses on practical support, breathing and relaxation techniques, interpersonal grounding and fostering an attitude of non-judgemental acceptance and openness around any arising experiences.

Integration focusses on active listening and awareness of non-verbal cues to focus and maintain participant's attention on their current emotional state as it evolves. The purpose is to focus the participant on emotional and experiential reprocessing of material relevant to depression that may have arisen in the treatment session. In this sense, integration is generalisable to both treatment conditions (psilocybin or placebo), because relevant material may arise in either. The relational model (that is, the model of the relationship between therapist and participant) builds on acceptance and commitment therapy (ACT) and compassion focussed therapy (CFT). This allows a flexible approach that responds to evolving content and dynamics of each session. All therapists in this trial will have a primary qualification in a related discipline and will have attended a three-day training programme developed by our lead therapist trainers. This programme comprises didactic teaching, group work and role play.

To further assess the psychological model, we may undertake a structured interview (SI) with participants before or after one or more of the psychological therapy sessions. This structured interview will be recorded and transcribed. We will collect objective data on the therapeutic

relationship via the STAR-P and STAR-C scales as described in the schedule of assessments. The quantitative and qualitative analysis of this data is not an intended outcome in this protocol.

5.1.4.18. Treatment

The treatment (psilocybin or placebo) will be delivered at V3.

5.1.4.19. Mini Neuropsychiatric Inventory v7.0 (MINI).

The MINI was designed as a brief, structured interview for the major Axis I psychiatric disorders in DSM-5 and International Classification of Diseases, version 10. Validation and reliability studies have been performed comparing the MINI to the Structured Clinical Interview for DSM-5 Patient Edition and the Composite International Diagnostic Interview (a structured interview developed by the World Health Organization). Version 7.0 of the MINI will be used for this study. The results of these studies show that the MINI has similar reliability and validity properties but can be administered in a much shorter period (mean 18.7 ± 11.6 min, median 15 min) than the above referenced instruments. It can be used by clinicians after a brief training session [2].

Relevant sections of the MINI will be administered at V1 for the purpose of confirming the diagnosis of MDD and ruling out psychiatric diagnoses that are exclusions for this trial. Diagnoses made using the MINI will be subject to clinical confirmation by a full psychiatric assessment.

5.1.4.20. 17 Item Hamilton Depression Rating Scale (HAM-D-17)

The HAM-D-17 is a 17-item scale used to measure the degree of symptom severity in depressed patients [3].

The HAM-D-17 will be administered at V1 and V2. The total score from this assessment will be used as eligibility criteria at screening and baseline (minimum total score of 14, in keeping with other similar TRD trials such as STAR*D and our own LQD trial). The structure of the interview will be standardised with the Structured Interview Guide for the HAMD (SIGH-D).

5.1.4.21. The Maudsley Staging Method (MSM)

The MSM implements a 15 point scale that scores various aspects that define treatment resistance [4]. Participants will score at least 5/15 on this scale to be eligible for the trial, with no upper limit on the degree of treatment resistance.

The MSM will be administered at V1.

5.1.4.22. Montgomery Asberg Depression Rating Scale (MADRS)

The MADRS is a clinician-rated scale measuring depression severity designed to be sensitive to change in clinical status. It consists of 10 items, each scored from 0 (normal) to 6 (severe), for a total possible score of 60. Higher scores denote greater severity.

The MADRS will be administered at V2, V5, V6 and V7. The structure of the interview will be standardised through the use of The Structured Interview Guide for the MADRS (SIGMA) [28]. The MADRS interview will be performed either by a member of the study team who will be blind to

treatment allocation and will not have attended the dosing session, or by a remote rater who is blind to treatment allocation and independent of the study team (funding dependent element). Regardless, before the interview commences, participants will be reminded not to discuss their beliefs about allocation with the interviewer and to concentrate purely on answers required for objective data collection.

5.1.4.23. The Columbia Suicide Severity Rating Scale (CSSRS)

The CSSRS is a clinician- and participant-rated scale that includes definitions of suicidal behaviour adapted from the Columbia Suicide History Form [6]. The definitions of ideation and behaviour were also used in the Columbia Classification Algorithm for Suicide Assessment, commissioned by the U.S. Food and Drug Administration to classify retrospective reports of potentially suicidal adverse events and to provide interpretable data to inform pivotal drug safety questions.

The CSSRS will be administered at V1, V2, V4, V5, V6 and V7.

5.1.4.24. Young Mania Rating Scale (YMRS)

The YMRS is an eleven-item multiple choice diagnostic questionnaire used to measure the severity of manic episodes [7]. The purpose of including this scale is to provide an objective measure in the event of a participant developing mania, either incidentally or as an adverse reaction.

The YMRS will be administered at V2, V4, V5, V6, and V7.

5.1.4.25. Scale to Assess the Therapeutic Relationship (Clinician) (STAR-C)

The STAR-C (and its participant-rated companion, the STAR-P) is a 12-item measure assessing the therapeutic relationship between patient and clinician on three components: Collaboration, Positive Clinician Input, and Emotional Difficulties (clinician version)/Non-Supportive Clinician (patient version) [8]. The Collaboration subtest reflects a good rapport and a shared understanding of goals, mutual understanding, openness, and trust. Positive Clinician Input reflects the perception (by the participant) of the clinician to encourage, support, and listen to the participant. Emotional Difficulties/Non-Supportive Clinician Input reflect problems in the relationship. The range of total scores for both versions is 0–48, with a higher score suggesting better therapeutic relationships. Each version of the scale takes approximately 5 minutes to complete. Total scores and subscale totals can be obtained.

We have added two further questions to this scale for therapists to indicate whether they have personal experience of the psychedelic state and whether they are open about this experience with participants. These items are optional and are intended to inform upon an analysis of whether prior personal experience of the psychedelic state in the therapist and their openness about this with the participant is a salient variable in determining therapeutic outcome. This is based upon observations in pre-prohibition literature that prior personal experience of the altered state of consciousness elicited by classical psychedelics was a prerequisite to be a therapist utilising them.

The STAR-C will be administered at V2, V5, V6 and V7.

5.1.5. Description of Participant Administered Measures

5.1.5.1. Quick Inventory of Depressive Symptomatology (QIDS-SR-16)

The QIDS-SR-16 is a 16-item self-rated scale designed to assess depression severity over time on nine diagnostic symptom domains [9]. The total score ranges from 0 to 27 with 0 representing no depression and 27 representing severe depression. The total score is the sum of the 9 symptom domains. The QIDS-SR-16 will be collected at V1, V2, V5, V6 and V7.

5.1.5.2. Generalised Anxiety Disorder Scale (GAD-7)

The GAD-7 is a self-rated screening and symptom severity measure covering 7 of the most commonly occurring anxiety symptoms, which are often comorbid with depressive symptoms [10]. Participants choose one of four severity scores and also indicate the degree to which these problems caused functional and/or social difficulties. Scores are derived from the values for each of the 7 domains.

The GAD-7 will be collected at V2, V5, V6 and V7. The GAD-7 takes less than 5 minutes to complete.

5.1.5.3. Childhood / Recent Traumatic Events Scale (CTS)

The CTS is a 7 item participant administered measure that collects data about childhood and recent traumatic life events and asks completers the extent to which the event was felt to be traumatic and the extent to which they confided in others about the event [11].

The CTS takes less than 5 minutes to complete and will be collected at V2 only.

5.1.5.4. State Trait Anxiety Inventory (STAI)

The STAI [12] is a 20 item likert scale designed to measure both state and trait facets of anxiety. It complements the GAD-7 by collecting data pertaining to longer term anxiety traits. Scores are derived from the state and trait question domains respectively.

The STAI takes less than 5 minutes to complete and will be collected at V2, V5, V6 and V7.

5.1.5.5. Schedule to Assess the Therapeutic Relationship (Participant) (STAR-P)

The STAR-P (and its clinician-rated companion, the STAR-C) is a 12-item measure assessing the therapeutic relationship between patient and clinician on three components: Collaboration, Positive Clinician Input, and Emotional Difficulties (clinician version)/Non-Supportive Clinician (patient version) [8]. The Collaboration subtest reflects a good rapport and a shared understanding of goals, mutual understanding, openness, and trust. Positive Clinician Input reflects the perception (by the participant) of the clinician to encourage, support, and listen to the participant. Emotional Difficulties/Non-Supportive Clinician Input reflect problems in the relationship. The range of total scores for both versions is 0–48, with a higher score suggesting better therapeutic relationships. Each version of the scale takes approximately 5 minutes to complete. Total scores and subscale totals can be obtained.

The STAR-P will be administered at V2, V5, V6 and V7. The STAR-P takes less than 5 minutes to complete.

5.1.5.6. Discontinuation Emergent Signs & Symptoms (DESS) Scale

The DESS is a scale designed to be sensitive to the signs and symptoms that develop on discontinuation of antidepressants, particularly those with monoaminergic reuptake inhibitor properties [13]. It is comprised of 43 items denoting various symptoms observed clinically in this scenario. Participants indicate whether each symptom is, since the last visit, a new symptom, an old symptom that has become worse, an old symptom that has improved, an old symptom that has not changed, or if the symptom is not present. The purpose of administering this scale is to objectively measure the degree and variability of antidepressant withdrawal symptoms in our study population, as this may predict drop out. The DESS will be collected at V2, only in participants who have been withdrawn from antidepressants. The DESS takes approximately 10 minutes to complete.

5.1.5.7. 5 Dimension Altered States of Consciousness Questionnaire (5D-ASC)

The 5D-ASC is a 94 item self-report measure of the acute effects of psychoactive drugs using 5 primary dimensions. Participants are given a statement pertaining to an element of conscious experience then asked to mark a point on a line between ‘No, not more than usually’ and ‘Yes, much more than usually’. The 5 dimensions are ‘oceanic boundlessness’, ‘anxious ego dissolution’, ‘visionary restructuralization’, ‘auditory alterations’, and ‘reduction of vigilance’ [14 29].

The 5D-ASC will be administered at V4. The 5D-ASC takes approximately 15 minutes to complete.

5.1.5.8. Altman Self-Rating Mania Scale (ASRM)

The ASRM is a 5-item self-report measure designed to probe the classical symptoms of mania with good test-retest reliability [15]. Participants are asked to choose one of four statements for each item.

The ASRM will be administered at V2, V5, V6 & V7. The ASRM takes less than 5 minutes to complete.

5.1.5.9. Symptom Check List (SCL-90)

The SCL-90 is a 90 item self-report measure [16], yielding nine scores along primary symptom dimensions and three scores among global distress indices. The primary symptom dimensions that are assessed are somatization, obsessive-compulsive, interpersonal sensitivity, depression, anxiety, hostility, phobic anxiety, paranoid ideation, psychoticism, and a category of "additional items" which helps clinicians assess other aspect of the participant’s symptoms (e.g. item 19, "poor appetite"). The three indices are global wellness index, hardiness, and symptom free. Numerous studies have been conducted demonstrating the reliability, validity, and utility of the instrument. It is one of the most widely used measures of psychological distress in clinical practice and research. The SCL-90 takes 12-15 minutes to complete and will be administered on V2 & V5.

5.1.5.10. Delayed Psychedelic After Effects Scale (DePAS)

The DePAS is an exploratory 20 item visual analogue scale measure designed for this trial that probes the presence of relative symptomatology across 10 domains that recreational users of psychedelics often report changes within. 2 statements per domain (that are phrased to be

conceptually and phenomenologically antithetical) are rated on a 10cm visual analogue scale with 5cm referring to 'about the same', 0cm 'much less' and 10cm 'much more'. The DePAS indirectly probes for symptoms of HPPD as well as both positive and negative experiences associated with psychedelic treatment. The DePAS takes less than 5 minutes to complete and will be collected on V4, V5, V6 and V7.

5.1.5.11. EuroQoL-5D-5L Scale (EQ-5D-5L)

The EQ-5D-5L was introduced by the EuroQoL Group in 1990. The EQ-5D-5L consists of a descriptive system and the EQ visual analogue scale with 5 levels (EQ VAS).

The descriptive system comprises five dimensions: mobility, self-care, usual activities, pain/discomfort and anxiety/depression. Each dimension has 5 levels: no problems, slight problems, moderate problems, severe problems and extreme problems. The participant is asked to indicate his/her health state by ticking the box next to the most appropriate statement in each of the five dimensions. This decision results in a 1-digit number that expresses the level selected for that dimension. The digits for the five dimensions can be combined into a 5-digit number that describes the participant's health state.

The EQ VAS records the participant's self-rated health on a vertical VAS, where the endpoints are labelled 'The best imaginable health state' and 'The worst imaginable health state'. The VAS can be used as a quantitative measure of health outcome that reflect the participant's own judgement.

The EQ-5D-5L will be obtained at V2, V6, and V7. The EQ-5D-5L takes less than 5 minutes to complete.

5.1.5.12. The Work and Social Adjustment Scale (WSAS)

The WSAS is a validated and accepted scale to assess the degree of functional impairment that directly results from a condition [18] The scale consists of 5 questions covering different functional domains that participants rate on an 8 point scale. The WSAS will be collected at V1, V2, V6 & V7 and takes approximately 1 minute to complete.

5.1.5.13. Tellegen Absorption Scale (TAS)

The TAS is a 34-item scale that assesses imaginative involvement and the tendency towards becoming mentally absorbed in everyday activities, which may predict response to the IMP(115). Participants rate the 34 items as either 'true' or 'false'. The total score is the number of 'true' ratings.

The TAS will be collected V2 and V7. The TAS takes approximately 5 minutes to complete.

5.1.5.14. Positive and Negative Affect Scale (PANAS)

The PANAS is a 20-item measure comprising 2 mood scales that measure different facets of positive and negative affect respectively [20]. Each item is rated on a 5 point Likert scale that is summed to deduce an overall score reflecting the tendency to experience positive or negative affective states.

The PANAS will be collected at V2, V4, V5, V6 and V7. We will collect the PANAS twice on the dosing day, before and after treatment. The PANAS takes less than 5 minutes to complete.

5.1.5.15. Self Compassion Scale - State (SCSS)

The self compassion scale is 26 item scale comprising 6 subsections that tend to load together. Self-compassion entails being kind and understanding toward oneself in instances of pain or failure rather than being harshly self-critical; perceiving one's experiences as part of the larger human experience rather than seeing them as isolating; and holding painful thoughts and feelings in mindful awareness rather than over-identifying with them. Results from the development of the scale indicate that self-compassion is significantly correlated with positive mental health outcomes such as less depression and anxiety and greater life satisfaction, thus this scale may provide important information about psychological mechanism [21]. We have modified this scale to reflect the need to collect state information about self compassion. The SCSS will be collected at V2, V4 and V7 and takes about 5 minutes to complete.

5.1.5.16. Self Compassion Scale - Trait (SCST)

The self compassion scale - trait is 26 item scale comprising 6 subsections that tend to load together. Self-compassion entails being kind and understanding toward oneself in instances of pain or failure rather than being harshly self-critical; perceiving one's experiences as part of the larger human experience rather than seeing them as isolating; and holding painful thoughts and feelings in mindful awareness rather than over-identifying with them. Results from the development of the scale indicate that self-compassion is significantly correlated with positive mental health outcomes such as less depression and anxiety and greater life satisfaction, thus this scale may provide important information about psychological mechanism [21]. The SCST will be collected at V2, V6 and V7 and takes about 5 minutes to complete.

5.1.5.17. Compassion Experience Scale (CES)

The CES is a 12-item likert scale developed in pilot form for this trial that explores the psychological theme of self-compassion experienced during treatment. Twelve items exploring elements of this are presented to participants, who are asked to indicate the degree of agreement on a line. These items are hypothesised to load onto 3 separate but related sub-themes: 1) self-kindness, 2) common humanity and 3) mindful experiencing. The scale is designed to capture data that may indicate whether experience of self-compassion is aetiologically relevant in understanding the putative mechanism of action of therapeutic change with psilocybin in depression. The CES will be collected at V4 only and takes less than 5 minutes to complete.

5.1.5.18. Trait Self-Description Inventory (TSDI)

The TSDI is a 50-item measure of the 'big five' personality traits [30]. Participants are provided with 50 statements and asked for their degree of agreement on a scale from 1 to 7 for stage 1 and 1 to 9 for stage 2.

The TSDI will be administered at V2 and V7. The TSDI takes approximately 10 minutes to complete.

5.1.5.19. Warwick Edinburgh Mental Wellbeing Scale (WEMBWS)

The WEMWBS is a 14 item inventory that measures mental wellbeing by asking participants to rate statements associated with different facets of wellbeing on a 5 point scale [22]. The scale has been validated for use in the UK population and takes less than 5 minutes to complete. The WEMBWS will be collected at V2, V6 and V7.

5.1.5.20. Connectedness to Nature Scale (CNS)

The CNS is a 14 item likert scale that measures facets of how connected raters feel to the natural world around them [23]. The scale has been validated for use in the UK populations and takes less than 5 minutes to complete. The CNS will be collected at V2, V6 and V7.

5.1.5.21. Short Assessment of Personality Scale (SAPAS)

The SAPAS is a very short assessment of personality [24] designed to capture indicative elements of personality that may further indicate personality structures captured within the ICD-10 and DSM-IV constructs of personality disorder. Eight items are rated 'yes' or 'no'. We will collect the SAPAS at screening in order to assess whether answers to any particular question are indicative of positive or negative response to psilocybin. The SAPAS will be collected at V1, V2 and V7 and takes 1 minute to complete.

5.1.5.22. Maudsley Treatment Inventory (MTI)

The MTI is a self-administered list of antidepressant and antidepressant augmentation treatments as defined by NICE Guidelines, the British National Formulary and the Maudsley Prescribing Guidelines (April 2018) along with space for dose, duration, tolerability, adherence and response [25]. It is a natural companion to the Maudsley Staging Method in classifying the degree of treatment resistance.

The MTI will be administered at V1 and takes approximately 10 minutes to complete.

5.1.5.23. THINC-it Cognitive Battery

THINC-it is a freely available, participant-administered, computerized tool integrating subjective and objective measures of cognitive function salient in adults with major depressive disorder (MDD). It is delivered as a computer program with simple instructions.

The THINC-it battery will be administered at V1, V2, V5 and V7 and takes approximately 10 minutes to complete.

5.1.5.24. Emotional Test Battery (ETB)

An ETB comprises a freely-available, participant-administered, computerized battery of tests for different facets of emotional processing. For example, the facial expression recognition test features six basic emotions (happiness, surprise, sadness, fear, anger and disgust) taken from the Pictures of Affect Series. The facial stimuli are presented on a computer screen (random order) for 500 msec and then replaced by a blank screen. Participants make their responses by pressing a labeled key on a computer keyboard. Each participant is asked to respond as quickly and as accurately as possible. In

the emotional categorization task, sixty personality characteristics selected to be extremely disagreeable (e.g., domineering, untidy, hostile) or agreeable (cheerful, honest, optimistic) are presented on the computer screen for 500 msec. These words are matched in terms of word length and ratings of frequency and meaningfulness. Participants are asked to categorize these personality traits as likable or dislikable as quickly and as accurately as possible. In the emotional memory task, immediately after completion of the emotional categorization task, participants are asked to recall as many of the personality traits as possible. This task therefore allows the assessment of incidental memory for positive and negative characteristics.

An ETB will be administered at V2, V5 and V7 and takes approximately 20 minutes to complete.

5.1.5.25. Maudsley Visual Analogue Scales (MVAS)

The Maudsley Visual Analogue scales indicate the quality of mood, experience of pleasure and experience of suicidal thoughts or feelings during a preceding interval of time that can be varied according to study need. These three items have been developed specifically to measure the three core symptoms that patients with TRD report most frequently. The MVAS may be administered at V1, V2, V3, V4, V5, V6 and V7 and will take less than 1 minute to complete.

6. Selection of Subjects

6.1. Inclusion Criteria

- Age 25 - 80 years
- Fluent in the English language
- Fulfil Diagnostic and Statistical Manual of Mental Disorders (5th Edition) (DSM-5) criteria for a primary diagnosis of current single or recurrent episodes of MDD of at least moderate severity but without psychotic features as defined on the MINI 7.0. Positive and primary diagnoses on the MINI 7.0 will be subject to confirmation at clinical interview by a psychiatrist.
- 17-item HAM-D score ≥ 14.
- Have failed to respond to 2 or more antidepressants prescribed at the minimum effective dose for at least 6 weeks OR at least 1 antidepressant prescribed at the minimum effective dose for at least 6 weeks AND a course of evidence-based psychotherapy given for at least 6 sessions.
- For those aged ≥ 60 years, the first episode of depression must have started prior to their 60th birthday.

6.2. Exclusion Criteria

- Diagnosis of bipolar disorder (defined as meeting DSM-5 criteria for bipolar 1 or bipolar 2) on the MINI 7.0. Positive diagnoses on the MINI will be subject to confirmation at clinical interview by a psychiatrist.
- Diagnosis of psychotic disorder (defined as meeting DSM-5 criteria for any psychotic disorder) on the MINI 7.0, EXCEPT substance/medication induced psychotic disorder where the duration was limited to the acute period of direct intoxication with the substance/medication. Positive diagnoses on the MINI will be subject to confirmation at clinical interview by a psychiatrist.
- Diagnosis of drug or alcohol dependence syndrome (defined as meeting DSM-5 criteria for any dependence syndrome) on the MINI 7.0. Positive diagnoses on the MINI will be subject to confirmation at clinical interview by a psychiatrist.
- Diagnosis of any personality disorder (defined as meeting DSM-5 criteria for any personality disorder) based on clinical interview and the MINI 7.0. Positive diagnoses on the MINI will be subject to confirmation at clinical interview by a psychiatrist.
- Diagnosis of any dementia (defined as meeting DSM-5 criteria for any dementia disorder) based on clinical interview by a psychiatrist.
- Personal history of a ≥ 1 suicide attempt in the past year requiring hospitalization, defined using the CSSRS (Q6 (past year) = “y”) and clinical interview with a psychiatrist.

- Other personal circumstances and behaviour judged to be incompatible with establishment of rapport or safe exposure to psilocybin.
- Depression secondary to other medical conditions
- Medical diagnosis incompatible with psilocybin treatment (see Section 6.2.1)
- Inability to provide a screening blood sample, urine sample or electrocardiogram.
- Biochemical abnormalities (defined as falling outside the normal reference range) as evaluated by a full blood count, full biochemistry profile and thyroid function tests. Biochemical abnormalities must also be determined as clinically significant by a medical doctor to fulfil the criterion for exclusion.
- Electrocardiographic abnormalities, defined as any abnormality that is not normal sinus rhythm and determined as clinically significant by a medical doctor.
- Women of child bearing potential not using adequate contraception (see Section 6.2.2).
- Pregnant or breast-feeding women.
- Those unable to give informed consent.
- Non-registration with a GP or failure to consent to sharing of the GP summary care record and any psychiatric assessments held.
- Those enrolled in another drug trial
- Hypersensitivity to the IMP or to any of the excipients or placebo

6.2.1. Exclusions for Pre-Existing Medical Conditions

Participants will be excluded if they have a current diagnosis of ≥1 of:

- Uncontrolled diabetes
- Hypertension (defined as a systolic blood pressure ≥ 160mm/Hg or a diastolic blood pressure ≥ 100mm/Hg on three separate readings). All readings of systolic blood pressure ≥ 140mm/Hg or diastolic blood pressure ≥ 90mm/Hg will be reviewed by a clinician. Hypertension ascertained prior to dosing will be subject to clinical confirmation via collateral information from the GP or other source.
- Cardiac failure, defined as class IV of the New York Heart Association classification
- Renal failure, defined as ≥ stage 4 (GFR ≤ 29mL/min)
- Liver failure, defined as a clinical diagnosis of liver fibrosis, cirrhosis of the liver, liver failure or advanced liver disease.
- Any cardiac arrhythmia, except atrial fibrillation.
- Any form of epilepsy

Past diagnosis of ≥1 of:

- Cerebrovascular accident or intracerebral trauma.

- Myocardial infarction within 1 year prior to the screening visit.

6.2.2. Women of Child Bearing Potential

A woman is considered of childbearing potential (WOCBP), i.e. fertile, following menarche and until becoming post-menopausal, unless permanently sterile. Permanent sterilisation methods include hysterectomy, bilateral salpingectomy and bilateral oophorectomy. A postmenopausal state is defined as no menses for 12 months without an alternative medical cause. A high follicle stimulating hormone (FSH) level in the postmenopausal range may be used to confirm a post-menopausal state in women not using hormonal contraception or hormonal replacement therapy. However, in the absence of 12 months of amenorrhea, more than one FSH measurement is required. A man is considered fertile after puberty unless permanently sterile by bilateral orchidectomy.

For females of childbearing potential who may participate in the study, the following methods of contraception, if used properly and used for the duration of the study, are considered sufficient:

- Oral, patch or injection combined or progestestogel only contraceptives. Hormonal contraception must be associated with inhibition of ovulation
- Intrauterine device
- Surgical sterilization
- Vasectomized partner provided that partner is the sole sexual partner of the WOCBP trial participant and that the vasectomised partner has received medical assessment of the surgical success.
- Sexual abstinence. Sexual abstinence is considered a highly effective method only if defined as refraining from heterosexual intercourse during the entire period of risk associated with the study treatments. The reliability of sexual abstinence needs to be evaluated in relation to the duration of the clinical trial and the preferred and usual lifestyle of the subject.

Periodic abstinence, i.e., calendar, symptothermal, or post-ovulation methods are not an acceptable form of contraception for this study.

Male participants will be required to use condoms. Male participants not willing to follow the contraception advice will be excluded.

The duration of contraception required is defined as until the end of the study.

The investigator and each participant will determine the appropriate method of contraception for the participant during the participation in the study. This will be documented at Screening (V1).

7. Assessment of Safety

7.1. Specification, Timing and Recording of Safety Parameters.

Patients with treatment resistant depression are considered a vulnerable population and therefore we will ensure monitoring and safety during the trial, as per GCP guidelines. Whilst the IMP is a Schedule

1, Class A substance, and therefore legally defined as having a high potential for harm and dependence, the summation of evidence reported here does not support this designation and we will be giving a single dose of the IMP only. We anticipate, therefore, that the risks associated with the IMP itself will be relatively low, however we comprehensively review the evidence for this in the Investigator's Brochure, and above in Section 5.3.

We do, however, consider that the risks associated with clinical depression itself, the preparations to receive the IMP, and with the consequences of randomisation, may be more pronounced than the risks of the IMP. These can be summarised under the risks consequent on the need to withdraw participants from their antidepressants prior to randomisation and the risk of further deterioration in mental state during the trial.

In the pilot trial we found that participants for the study were content to discontinue their current antidepressant medications because, by definition, they were inefficacious +/- had burdensome side effects. There was no instance in which a participant, as a consequence of medication withdrawal, demonstrated a deterioration in mental state severe enough to warrant clinical concern about risk to self or others. However, this may in part have been because all participants knew that they would be receiving the full dose of the IMP, whereas in this study participants will be aware that they will be randomised. This has two potential effects:

1) Affecting recruitment
2) Affecting a participant's mental state during and after the Dosing Visit (V3)

Assessing these parameters is a necessary part of the measurement of feasibility in this study, so we will record drop outs and reasons in the CRF and we will also arrange for participants to restart antidepressant therapy in liaison with their primary or secondary care teams and/or refer them for psychological therapy if their mental state worsens. One participant dropped out immediately after receiving psilocybin in the pilot trial, citing a lack of efficacy and refusing to engage in further follow up. This participant did subsequently provide follow up data six months later.

The screening process will exclude participants who, because of their prior history, may react more unfavourably to the IMP. A list of exclusion criteria is contained in Section 6.2. Medical comorbidities will be assessed by a medical history, physical examination, routine clinical laboratory investigations and an electrocardiogram. Final eligibility will be determined by an experienced clinician. The medical history, physical examination, clinical laboratory investigations and an electrocardiogram are performed with the primary purpose of excluding participants with medical conditions that may be incompatible with treatment with psilocybin, rather than because of concerns about the toxicity of the IMP itself. For example, it would be inappropriate to include a participant with uncontrolled diabetes in the trial, because the effect of the drug may include appetite suppression, which may adversely affect glucose levels. Further physical examination and/or clinical laboratory investigations may be initiated by the study clinician at their discretion at any point during the follow up period, and would be particularly appropriate if, for example, an adverse event is reported where the aetiology may be clarified by further such investigation.

The SP phase will, for a proportion of participants, involve stopping established antidepressant regimens. Participants will be supported by a psychiatrist and the study team during this period. Discontinuation syndrome signs and symptoms will be captured with the DESS scale. Drop outs due to intolerance of antidepressant withdrawal and/or worsening of mental state will be recorded and reported in line with this protocol. We have specified a variable period for the SP phase, particularly in view of the need for clinical flexibility during this time. We will allow prescription of symptomatic medications as described above to manage the discontinuation syndrome and the insomnia that can result from withdrawal of antidepressants. However, participants will need to have refrained from using these (aside from short acting hypnotics) for 24 hours prior to the morning of the Dosing Visit (V3).

We will specifically enquire about adverse events at each of the follow up visits. Adverse events will be enquired about with open ended questions initially, however we will also use the UKU schedule for side effects to psychotropics to structure collection of data with closed questions enquiring for specific adverse events. Adverse events of special interest are further described below. All adverse events will be recorded.

Participants will be asked to contact the study team about any adverse events they experience in-between face to face visits. A member of the team will speak to the patient and trial clinicians will advise and assess the patient’s health and wellbeing. Adverse events will be reported as outlined in Section 9.2.

Safety data will be made available to the DMEC and TSC, who will then advise the Sponsor whether the trial should continue or be terminated on the basis of the data provided.

7.2. Procedures for Recording and Reporting Adverse Events

We will follow the relevant definitions provided by The Medicines for Human Use (Clinical Trials) Regulations 2004 and Amended Regulations 2006 with regards to adverse events:

Adverse Event (AE): Any untoward medical occurrence in a subject to whom a medicinal product has been administered including occurrences which are not necessarily caused by or related to that product.

Adverse Reaction (AR): Any untoward and unintended response in a subject to an investigational medicinal product which is related to any dose administered to that subject.

Unexpected Adverse Reaction (UAR): An adverse reaction the nature and severity of which is not consistent with the information about the medicinal product in question set out in the Investigator's Brochure

Serious Adverse Event (SAE), Serious Adverse Reaction (SAR) or **(Suspected) Unexpected Serious Adverse Reaction ((S)USAR)**: Any adverse event, adverse reaction or unexpected adverse reaction, respectively, that

- Results in death;

- Is life-threatening;
- Required hospitalisation or prolongation of existing hospitalisation;
- Results in persistent or significant disability or incapacity;
- Consists of a congenital anomaly or birth defect.

Although not a serious adverse event, any unplanned pregnancy will also be reported via the SAE reporting system.

7.2.1. Reporting Responsibilities

King's College London and South London and Maudsley NHS Foundation Trust (the co-sponsors) have delegated the delivery of the Sponsor's responsibility for Pharmacovigilance (as defined in Regulation 5 of the Medicines for Human Use (Clinical Trials) Regulations 2004 to the King's Health Partners Clinical Trials Office (KHP-CTO).

All SAEs, SARs and SUSARs will be reported immediately (and certainly no later than 24 hours) by the Investigator to the Chief Investigator who will review and report to the KHP-CTO in accordance with the current Pharmacovigilance Policy.

The KHP-CTO will report SUSARs to the regulatory authorities (MHRA, competent authorities of other EEA (European Economic Area) states in which the trial is taking place.

The Chief Investigator will report to the relevant ethics committee. Reporting timelines are as follows:

- SUSARs which are fatal or life-threatening must be reported not later than 7 days after the sponsor is first aware of the reaction. Any additional relevant information must be reported within a further 8 days.
- SUSARs that are not fatal or life-threatening must be reported within 15 days of the sponsor first becoming aware of the reaction.
- The Chief Investigator and KHP-CTO (on behalf of the co-sponsors), will submit a Development Safety Update Report (DSUR) relating to this trial IMP, to the MHRA and REC annually.

7.2.2. Documentation, Classification & Follow Up of Adverse Events

AEs occurring after the participant signs the consent form until the last study visit will be recorded. AEs that occur prior to the Dosing Visit (V3) and after the screening visit (V1) will be recorded in the adverse event log, with the causality set to '1' (not related). We will not consider diseases or conditions present before the treatment period to be AEs unless they worsen after the Dosing Visit (V3). All significant illnesses in the 3 months prior to the screening visit (V1) as well as all illnesses ongoing at the screening visit (V1) should be recorded in the case record form.

All clinical laboratory results, vital signs, and ECG results or findings will appraised by the investigator to determine their clinical significance. Isolated abnormal clinical laboratory test results, vital sign findings, or ECG findings (i.e., not part of a reported diagnosis) should be reported as AEs if they are symptomatic, require corrective treatment, or constitute an AE in the investigator's clinical judgment.

At each time point, the investigator will determine whether any AEs have occurred by evaluating the participant. AEs may be directly observed, reported spontaneously by the participant or by questioning the participant at each time point. The investigator must assess all AEs to determine intensity, causality and seriousness, in accordance with the definitions in Sections 9.1 and 9.2. The investigator's assessment must be clearly documented in the study site's source documentation. Diagnosis should, in preference, be specified as the AE or SAE term. If a diagnosis is unavailable, then the primary sign or symptoms should be reported with additional narrative details.

Each AE will be classified according to the following criteria:

- Mild. The AE does not interfere in a significant manner with the participant's normal level of functioning.
- Moderate. The AE produces some impairment of functioning but is not hazardous to the participant's health.
- Severe. The AE produces significant impairment of functioning or incapacitation and is a definite hazard to the participant's health.

Severity versus Seriousness: Severity is used to describe the intensity of a specific event. However, the event itself may be of relatively minor medical significance (such as severe headache), and therefore not serious, which reflects the overall impact on the participant.

When changes in the intensity of an AE occur more frequently than once a day, the maximum intensity for the experience should be noted. If the intensity category changes over several days, those changes should be recorded separately (with distinct onset dates).

Each AE will be assessed as to its relationship to the IMP, based on the following criteria. Although the attribution by the investigator will be collected for reported events, for analytic purposes a temporal association with the use of the IMP will be assumed sufficient for at least plausible association.

- Not related. No causal relationship exists between the IMP and the AE, but an obvious alternative cause exists, for example the participant's underlying medical condition or concomitant therapy.
- Possibly related. A connection with the administration of the IMP appears unlikely but cannot be ruled out with certainty. An AE may be considered possibly related if or when it meets 2 of the following criteria:
  - (1) it follows a reasonable temporal sequence from administration of the IMP;
  - (2) it could not readily have been produced by the participant's clinical state, environmental or toxic factors, or other modes of therapy administered to the participant; or
  - (3) it follows a known pattern of response to the IMP.
- Related. It is reasonable and plausible that the AE has been caused by the IMP.

The relationship of the AE to the IMP will be further considered according to the following criteria:

- Known class effect
- Biological plausibility
- Lack of alternative explanation

The **action** taken regarding the IMP in relation to the AE can generally be divided into two categories

1. Dose not changed. No change in the study drug dosage was made.
2. Not applicable. Participant died, study treatment had been completed prior to reaction/event, or reaction/event occurred prior to start of treatment

Other actions taken for an AE may be classified as follows

1. None. No treatment was required.
2. Medication required. Prescription and/or over-the-counter medication was required to treat the AE.
3. Hospitalisation or prolongation of hospitalisation required. That is, hospitalisation was required or prolonged because of the AE, whether medication was required or not.
4. Other

The **outcome** of an AE may be classified thus:

1. Recovered/resolved. That is, the participant fully recovered from the AE with no residual effect observed.
2. Recovering/resolving. That is, the AE improved but has not fully resolved.
3. Not recovered/not resolved. That is, the AE itself is still present and observable.
4. Recovered/resolved with sequelae. That is, the residual effects of the AE are still present and observable, including sequelae/residual effects.
5. Fatal. That is, when death is a direct outcome of the AE.
6. Unknown

All AEs will be followed up until resolved or stable. The outcome will be documented on the CRF.

7.2.3. Other Procedures for Reporting and Recording Adverse Events

Due to the limited safety information available for psilocybin, in addition to reporting all adverse events in line with the requirements of this protocol, the study team will be recording adverse events separately. These AEs will be transferred to Worldwide Clinical Trials for adding into a safety database, managed by both Worldwide Clinical Trials and COMPASS Pathways, allowing for the continued development of safety data around psilocybin. Patients will be asked to consent for this aspect of the research; patients who do not consent will not be excluded from study participation.

For the purposes of the safety database maintained by Compass Pathways and Worldwide Clinical Trials, they will be informed of the unblinding results at the end of the trial (the time point of database lock).

7.3. Adverse Events of Special Interest (AESI)

An adverse event of special interest (AESI) is an AE (serious or non-serious) of scientific and medical concern specific to the IMP. Such AEs may require further investigation to characterise and understand them.

The following events, not occurring during the Dosing Visit (V3) or open label extension dosing (OLE2), are considered to be AESIs

- Mania
- Psychosis
- Suicide attempt or self-harm
- Hallucinogen persisting perception disorder

AESIs will be specifically enquired about during follow up visits and captured both narratively, as AEs and by formal scales, for example the HPPDS, DEPAS, AMRS and YMRS.

7.4. Adverse Events That Do Not Require Reporting

Since the IMP does not have a license or SmPC, all adverse events will be reported. Adverse events will be reported from the start of Dosing Visit (V3) until completion of follow up or until the end of the open label extension if the participant has consented to this, whichever is longer.

7.5. Treatment Stopping Rules

The trial may be prematurely discontinued by the Sponsor, Chief Investigator or Regulatory Authority on the basis of new safety information or for other reasons given by the Data Monitoring & Ethics Committee / Trial Steering Committee regulatory authority or ethics committee concerned.

If the trial is prematurely discontinued, active participants will be informed and no further participant data will be collected. The Competent Authority and Research Ethics Committee will be informed within 15 days of the early termination of the trial.

8. Biological Sample Analysis

Analysis of biological samples beyond those required for safety is not an intended outcome of this project and future ancillary analyses for -omics will be subject to further authorisations

9. Data Management & Confidentiality

Research data will be identified by a unique code and participant initials with restricted access. Electronically captured data will be time-stamped and changes to individual data points will be recorded. Electronic data will be backed up regularly via an encrypted connection to a secure server with second site resilience. We will adhere to NHS confidentiality practice, and to the Research Governance Framework in monitoring and managing the research. As CI, Professor Young will undertake overall responsibility for management of the project. The Data Management Plan details measures taken to promote data quality, for example, range checks for data values input into the eCRF.

The Sponsor will have access to the final dataset, with no limits on access for investigators. The chief investigator will be the principal custodian of the data. The Statistical Analysis Plan will be made available via the Sponsor's websites.

10. Ancillary and Post Trial Care

At the end of the trial care of participants will be handed back to referrers.

The trial is joint Sponsored by an NHS Trust and therefore participants will have access to compensation schemes under this remit. King's College London, as joint Sponsor, has obtained insurance which provides no-fault compensation, i.e. for non-negligent harm.

11. Authorship Eligibility

All study team members who make a substantial contribution to the trial will be listed as authors or acknowledged in the manuscript. We do not intend to use professional writers.

INFORMED CONSENT FORM

A randomised, placebo-controlled trial of psilocybin in treatment resistant depression: a feasibility study

Participant Identification Number for this Study:____________________

Please read the following and **write your initials in each box** before writing your full name, signing and dating at the bottom of the form.

1	I confirm that I have read and understood the participant information sheet, dated 11 MAR 2021, relating to the above trial. I have had the opportunity to think about the information, ask questions and have had these answered satisfactorily. I understand why the research is being performed and any risks involved.		<- Initials
2	I understand that my participation is voluntary and that I am free to withdraw at any time without giving a reason, without my medical care or legal rights being affected.		<- Initials
3	I give permission to the study team to contact my GP and/or mental health team in order to collect additional information about my history. I give permission to the study team to inform my GP and/or mental health care team about my progress. I understand that this information will be kept confidential. I understand that after my participation in the study has ended that my care will be passed back to my usual care team.		<- Initials
4	I understand that I will need to provide the contact details of a trusted friend or relative to be eligible for the study. I consent to the study team speaking with this person about my involvement and progress through the trial, and in the event of a deterioration in my mental health, or an emergency.		<- Initials
5	I understand that I will need to stop taking my current antidepressant treatments to be eligible for this trial. I understand that the study team and caregiver (if I have one) will need to closely monitor me during this time. If I have a caregiver, then I understand that the study team will need to make contact with me at least on a weekly basis. If I do not have a caregiver, then I understand that the study team will need to make contact with me at least every 4 days.		<- Initials
6	I understand that one outcome of my participation in this trial is that my condition could significantly worsen. In this instance, I understand that the study team would usually recommend that I restart on the best available treatment.		<- Initials
7	Notwithstanding (2), I understand that, once I have swallowed the study treatment on the Dosing Day, I will be required to remain in the Clinical Research Facility at King's College Hospital until the study team tell me that I can leave. This means that the study team may require me to stay in the hospital overnight if they think I am not safe to go home after the treatment. Moreover, I understand that the study team may, in the least restrictive manner, prevent me from leaving the Clinical Research Facility at King's College Hospital if they think I would be a risk to myself or others. I understand the reasons for this and give my explicit permission for such steps to be taken in my best interests, if necessary.		<- Initials
8	I understand and consent to blood samples and urine samples being taken and analysed for the purposes of routine laboratory tests to determine my eligibility for this study.		<- Initials
9	I consent to an additional 5 (five) blood samples being taken during the course of the study. I understand that these samples will be frozen and stored in a secure location with my personal identifying data removed. I understand and consent to these samples being used for biological research in the future. I understand that I will not benefit from this research directly. I understand that no research performed with these samples will ever identify me personally without my explicit further consent. I understand that this part of the study is optional.		<- Initials
10	I consent to my study visits being recorded by audio and/or visual recording equipment if necessary. I understand that I can request for the recording equipment to be switched off and the study team will do this unless my request contravenes requirements laid down by relevant legislation. I understand that this data will be subject to the same security processes regarding storage, use in research and destruction as the rest of my personally identifying research data.		<- Initials

11	I understand that my personal identifying data will be stored by the research team in secure clinical databases, which may include the sponsoring NHS Trusts' electronic record. I understand that this personal identifying data will not be shared with anyone outside of the research team or clinical team without my further consent.		<- Initials
12	I consent to my deidentified data (data without attached personal identifying data) being entered into relevant study databases. I understand how the information will be collected and stored. I understand that the study information will be stored for as long as it may be usefully used in research and, in any event, for a minimum of 15 years.		<- Initials
13	I consent to my deidentified data to be used for other research and training in the future. I understand that this data may be shared anonymously with other researchers. I understand that the research team may use direct, anonymised quotes that will not identify me personally for the purposes of future training related to this treatment, and other research. I understand that any data that may identify me personally will <u>not</u> be shared without my further consent being sought.		<- Initials
14	I understand and consent to sharing step counts and sleep data collected by my own personal fitness tracker or smart phone for the duration of my inclusion in this study. I consent to the use of my deidentified data collected from any or all of these devices to be used for research, subject to the restrictions outlined in items 9 and 10 of this consent form. I understand that this part of the study is optional and I do not have to consent to this to be considered eligible for this study.		<- Initials
15	I consent to be contacted in the future regarding follow up studies. The research team may request my participation in similar studies or other related medical research studies. I understand that I do not have to consent to these studies or to this item on this consent form in order to be considered eligible for this study.		<- Initials
16	I know how to contact the research team if I need to, and how to get information about the results of the research.		<- Initials
17	I understand that data collected during the study may be looked at by individuals from the Research Team (at the Institute of Psychiatry, Psychology & Neuroscience, King's College London), by regulatory authorities, representatives from the Sponsor or from the NHS Trust, where it is relevant to my taking part in this research. I give permission for these individuals to have access to my data.		<- Initials
18	I consent to my safety data, which may include instances of my medical history, being transferred and stored in a safety database held securely by Worldwide Clinical Trials and COMPASS Pathways. I understand that this data will be anonymised. I understand that this data may be retained indefinitely by COMPASS Pathways following completion of the trial. In regard to data held in this safety database, I understand that I am able to request the following: 1 Review my data held in the safety database. 2 Request for any inaccuracies to be corrected. 3 Raise objections to its content and to the continued processing of my data in this database. 4 Request to have my data removed from this database.		<- Initials
19	I understand that by signing this consent form that I am <u>not</u> necessarily eligible to receive the study treatment. I understand that the process of determining my eligibility for this study is ongoing and I understand that the study team will not be able to confirm my eligibility until the baseline visit, shortly before the dosing visit.		<- Initials
20	I agree to take part in the above study		<- Initials

↑Name of Participant↑	↑Date↑	↑Signature↑
↑Name of Person Taking Consent↑	↑Date↑	↑Signature↑

<u>Original copy: Store in the investigator file</u>

<u>Give one copy to the participant</u>

PsiDeR Trial
DATA MANAGEMENT PLAN

12. Introduction

The PsiDeR trial collects data in different formats. In designing the data collection strategy we have attempted to balance the inherent advantages and disadvantages of paper and electronic data capture methods. More broadly, the PsiDeR trial is a feasibility trial, and thus unlikely to be used for commercial licensing. Nonetheless, data integrity and ease of collection for both participant and researcher were considered important in this trial.

We consulted extensively over the data collection strategy, both informally with colleagues in different departments and also more formally with the members of our Trial Steering Committee and the King's Clinical Trials Office. More particularly, we asked appropriately qualified members of different departments to 'destructively test' versions of our electronic data capture system to expose weaknesses that were then corrected before submission to the ethics committee.

The inherent advantages of electronic data collection (imposition of data validity limits, convenience of access and ease of collation of data for analysis) were balanced against the disadvantages (high cost of fully auditable systems, low convenience of fully auditable systems for participants and researchers, danger of data loss with MS Excel solutions, danger of data corruption or unauditable data changes with MS Excel solutions).

The PI considered the above over multiple meetings with Ben Carter and Hassan Jafari at the Biostatistics unit and via email and a formal meeting with Helen Critchley and Matt Simpson at the King's CTO. Our data collection strategy was also discussed with PPI representatives sitting on our Trial Steering Committee who also have appropriate technical experience (Sarah Markham and Michael Bourne). The agreed strategy, as described below, was developed principally by James Rucker and destructively tested by Hasan Jafari and Matt Simpson.

13. Data Collection Strategy

13.1. Paper Forms

Paper clinical record forms will be used for the following:

1. Wet ink medic signatures to confirm informed consent at screening and participant eligibility at each face to face visit.
2. The MINI version 7 (screening)
3. The MADRS. The rationale for this is that we will ask an independent researcher to collect this outcome data to achieve objectivity and this will be easier to achieve with a paper scale.

Data from the MADRS will transferred into the equivalent form of the database and the original kept as source

13.2. Electronic Database

13.2.1. Microsoft Excel

A Microsoft Excel solution was designed with destructively tested VBA code. This implements the following features:

1. Cell locking and password protection. Only cells in which data should be entered are unlocked for editing. All other cells are locked with a password that only the study team know.
2. Data validation. Each cell where data can be entered is programmed with data validation limits that prompt the user if the data falls outside these limits. For example, for an outcome measure where the only valid answer are the numerals 1-5, the user will be prompted with an error message if they type ‘0’ or ‘6’ or ‘A’, say.
3. Completion validation. Each cell where data can be entered is automatically coloured red. When a valid data point is entered, the red colour disappears. This allows easy checking for missing data by the study team. A synopsis sheet at the start of each visit is programmed to indicate when all data points on a given scale have been completed, in a similar manner.
4. Data logging. Each change in a cell is logged in a text file stored separately to the Excel solution. This text file lists, line by line, the date and time, username, cell location, old value and new value for each change.
5. A consolidated data sheet showing a unique ID for each data point collected as well as the data value is collated automatically at the end of each excel workbook. This can be printed, signed, dated and stored as a further source record.
6. Remote backups. The laptop used for data collection stores remote backups to an encrypted, network attached server within the KCL firewall. This has the effect of recording multiple backups of each instance of each visit for each participant over time. This will allow changes over time, in addition to the verbose data logging, to be analysed if necessary.
7. Paper backups. The entire Excel solution is designed so that it can be printed and hand filled as a backup. Paper forms will be kept as source data if this is the case.

Collection via this medium was agreed for the following forms

1. The case record form for each visit (except for wet-ink signed documentation of consent and eligibility)
2. Researcher rated scales - HAM-D-17, MSM, CSSRS, YMRS, MTI and STAR-C

3. Participant rated scales - QIDS-SR-16, GAD-7, CTS, STAI, STAR-P, DESS, ASRM, SCL-90, EQ-5D-5L (Items 1-5), WSAS, TAS, PANAS, SCSS, SCST, CES, TSDI, WEMWBS, CNS, SAPAS.

MS Excel is not suitable for collecting visual analogue scale data. We investigated solutions for this and decided on the Python Scripting GUI PsychoPY for visual analogue scales, implemented on the same laptop used for other data collection. PsychoPY implements automatic data logging and data validity protocols as standard. Routines were designed to collect the following data.

1. Participants rated scales - MVAS, EQ-5D-5L (item 6 only), 5D-ASC, DePAS, ETB.
2. The ETB will be collected using a touchscreen to ensure consistency of response between participants. The other scales do not necessarily require a touchscreen, although we may use one for convenience.

Data from PsychoPy will be stored in the folder as the rest of the participant's data for that visit. PsychoPY generates both log files and timestamped Excel spreadsheets of data, so there will be an audit trail of data entry. The data will be backed up in the same way as the rest of the data.

The THINC-it cognitive test is administered using a freely available programme downloadable from the developers, which will be administered using the same touchscreen laptop as above to ensure consistency between participants. Data from THINC-it will be stored in the folder as the rest of the participant's data for that visit. THINC-it generates log files of data entry, so there will be an audit trail. The data will be backed up in the same way as the rest of the data.

14. Data Storage

Paper scales will be stored in ring binders with one ring binder per participant. Whilst participants are actively involved with the trial these will be stored in the Clinical Research Facility, which is accessible only by swipe card. All research data will be identified by a participant ID code. No personally identifiable identification data will be stored in individual participant's ring binders.

Paper data that may contain sensitive information, such as GP summary care records and psychiatric assessments, will be stored in a separate binder in a locked office.

Electronic data will be stored on an encrypted study laptop. The laptop will be connected to the WiFI network within the KCL firewall. We will then backup research data to a secure server located in a locked office. SharePoint will be used (as advised by KCL IT) for second site resilience of electronic data. As a final backup and to ensure a reasonable audit trail. Electronically captured data and associated log files can be printed and stored with the rest of the paper research data for each

participant when they have finished their involvement in the trial. This paper record can then be stored in a locked office.

Personally identifiable data will be stored along with the linking ID in a separate, password protected Excel workbook stored on the study laptop, the secure remote server and SharePoint. This file will only be accessible by members of the research team and will not be shared with external agencies unless legally required or for pharmacovigilance processes surrounding serious adverse events or SUSARs.

14.1. Data Validation, Database Creation & Database Lock

Data validation will be ongoing through the trial. The electronic database imposes mandatory limits on data points to ensure validity and checks for missing data in scales automatically, generating a live warning at each visit if this is detected. The electronic database can be collated and interrogated to generate reports for the DMC.

The data logger will contain details of any user changes that have been made, to allow discrepancies to be queried and audited.

A database containing all trial data points will be generated after the final visit for the final participant. This will include a unique ID for each piece of data that identifies the participant (by study ID), the visit number, the scale, the specific data point and a numeric code that identifies the date of completion. The other data points will identify the possible input values for each data point and the data point itself. Null data points will be recorded as zero length text strings.

Database lock will be signed and confirmed by the PI and trial senior statistician. The data management plan will be reviewed by the DMEC at six monthly intervals and final database approval will be independently authorised by the DMEC chair, who is independent of the trial sponsor and study team.

15. Data Sharing

The Trial Management Group will consider data sharing with external researchers on receipt of a distinct Statistical Analysis Plan that is approved by the Trial Management Group.

Data Monitoring Commitee
Charter for Trial Steering Committee (TSC) acting also as a Data Monitoring Committee (DMC)

Study/ Trial full Title:	Psilocybin in Depression Resistant to Standard Treatments
Protocol short title:	Psilocybin in Depression Resistant to Standard Treatments (PsiDeR)
EudraCT Number:	2018-003573-97
IRAS Project ID:	252750
REC No:	20/LO/0206
Investigational product:	Psilocybin 25 mg
Chief investigator:	Professor Allan Young
Principal Investigator:	Dr James Rucker
Sponsor name:	King's College London/South London & Maudsley NHS Foundation Trust

Version History:

Version 0.1 – Draft	26th February 2019
Version 0.3	27th February 2019
Version 1.0	16th January 2020

Version 1.0, 16th January 2020

(Developed using Advice and information on the role and procedures for setting up a DMC, DAMOCLES Study Group: A proposed charter for clinical trial data monitoring committees: helping them do their job well. Lancet – 2005; 365: 711-722)

Prepared by: Hassan Jafari (Trial statistician)

16. Introduction

The role of the trial steering committee (TSC) and Data Monitoring Committee (DMC) is to provide overall supervision for the PsiDeR trial on behalf of the Trial Sponsor and the Trial Funder and to ensure that the trial is conducted according to the guidelines for Good Clinical Practice (GCP), Research Governance Framework for Health and Social Care and all relevant regulations and local policies.

The background to PsiDeR trial, its objectives, assessments, interventions, etc, are described in the study/trial protocol.

17. Roles and responsibilities

17.1. A broad statement of the aims of the TSC/DMC

The purpose of this document is to describe the roles and responsibilities of the independent TSC/DMC for the PsiDeR trial, including the purpose and timing of meetings, methods of providing information to and from the committee, frequency and format of meetings, statistical issues and relationships with other committees.

To provide overall supervision of the trial independently of the investigators. They will also safeguard the interests of trial participants, potential participants, their families, their carers, investigators, and the sponsor; to assess the safety of the intervention during the trial, and monitor the trial's overall conduct, and protect its validity and credibility.

17.2. Terms of reference

- To provide advice, through its Chair, to the Trial management Group (TMG), the sponsor and the trial Funder on all aspects of the trial.
- To monitor and supervise the progress of the trial towards its overall objectives, review accrual and results of the trial, adherence to the protocol, patient safety and the consideration of new information of relevance to the trial and the research question.
- To ensure that the rights, safety and well being of the trial participants are the most important considerations and should prevail over the interests of science and society.
- To ensure that all relevant approvals are obtained before a project begins.
- To agree proposals for substantial protocol amendments and provide advice to TMG regarding approvals of such amendments.
- The TSC should inform the Funder if:
    - There are concerns about the safety of the participants.
    - Accrual is too low to provide meaningful results.
    - It is evident that if the study continues, it would fail to provide a clear benefit.
- To recommend whether to continue or terminate the study or further adapt it based in safety considerations.

17.3. Specific roles of the committee

To undertake interim review of the trial's progress by:

- Assessing data quality, including completeness
- Monitoring recruitment figures and losses to follow-up
- Monitor evidence for treatment harm
- Monitoring compliance with the protocol by participants and investigators
- Advising on protocol modifications suggested by the TMG
- Monitoring continuing appropriateness of patient information
- Considering ethical implications
- Assessing the impact and relevance of external evidence
- Maintaining confidentiality of all trial information that is not in the public domain
- Protecting validity and scientific credibility of the trial

18. Before and early in the trial

18.1. Whether the committee will have input into the protocol

All potential members should have sight of the protocol/outline before agreeing to join the committee. Before recruitment begins the trial will have undergone review by the funder, scrutiny by other trial committees and the Research Ethics committee.

Therefore, if a potential member has major reservations about the trial (e.g. the protocol or the logistics) they should report these to the CI or trial coordinating team and may decide not to accept the invitation to join. Committee members should be independent and constructively critical of the ongoing trial, but also supportive of aims and methods of the trial.

18.2. Will the committee meet before the start of the trial?

The committee will meet as soon as the trial starts to discuss the protocol, the trial, any analysis plan, future meetings, and to have the opportunity to clarify any aspects with the CI and PI and coordinating team.

18.3. Any specific regulatory issues

The committee will be aware that this trial is a Clinical Trial of an Investigational Medicinal Product (CTIMP) and therefore does require a Clinical Trials Authorisation from the Medicines and Healthcare

products Regulatory Agency and is governed by the UK legislation enacting the European Union Clinical Trials Directive.

18.4. Whether members of the committee will have a contract

Members will not formally sign a contract but should formally register their assent to join the group by confirming (1) that they agree to be on the committee and (2) that they agree with the contents of this Charter. Any competing interests should be declared at the same time. Members should complete and return the form in Annexe 1. Observers attending any part of the meeting should sign a confidentiality agreement on the first occasion they attend all or part of a meeting (Annexe 2).

19. Composition and primary responsibilities of the TSC/DMC

The PsiDeR TSC is a multidisciplinary group comprising of the following members who jointly have responsibilities for the design, conduct and evaluation of the clinical research project.

The members of the committee are independent of the trial (e.g. are not involved with the trial in any other way and will not have competing interests that could impact on the trial). Any competing interests, both real and potential, will be declared in Annex 1.

19.1. Agreement

TSC members should formally register their agreement to be a member of the committee as well as their agreement with the contents of the charter, trial confidentiality and should declare any potential conflicts of interest.

Independent and non-independent members should complete and return a signed agreement and competing interests form provided at the end of this charter (Annex 1 and Annex 2).

Table 1. Composition of TSC/DMC for PsiDeR

Name	Role	Independent/ non-independent	Executive member
Professor Celia Morgan	Chair	Independent	Yes
Professor Allan Young	Chief investigator	Non-independent	No
Dr James Rucker	Principal investigator	Non-independent	No
Dr Ben Carter	Trial statistician	Non-independent	No
Winston Banya	Statistician	Independent	Yes

Tim Mantingh	Trial Assistant	Non-independent	Yes
Hassan Jafari	Statistician	Non-independent	Yes
PPI rep 1	Representative of relevant patient group	Independent	Yes
PPI rep 2	Representative of relevant patient group	Independent	Yes
PPI rep 3	Representative of relevant patient group	Independent	Yes
PPI rep 4	Representative of relevant patient group	Independent	Yes
PPI rep 5	Representative of relevant patient group	Independent	Yes

19.2. Responsibilities

The TSC/DMC on behalf of the Sponsor and Funder will have overall responsibility for the design and conduct of the trial and safeguarding the rights, safety and wellbeing of the participants.

Responsibilities of the TSC/DMC to include:

- Reviewing selection/recruitment/retention of participants.
- Finalising and reviewing study protocol and other study documentation.
- Determining if amendments to the protocol or changes to study conduct are required and deciding on changes to these and to study conduct in general. Any changes to trial documentation or conduct must be notified to the TSC.
- Reviewing adherence to the protocol by the investigators and participants.
- maintain confidentiality of all trial information that is not already in the public domain
- Assessing the impact and relevance of the external evidence.
- Assessing the integrity and completeness of the data collected.
- Monitoring the overall conduct of the trial, ensuring that it follows the standards set out in the guidelines of GCP, assessing the safety of the intervention, recruitment figures and completion of the trial assessments and follow-ups.
- Reviewing, commenting and making decisions on extension requests.

- Reviewing the safety recommendations of the trial statistician and/or other study committees and suggesting appropriate action to the TMG.
- Monitoring the progress of the trial and deciding on appropriate action in order to maximise the chances of completing it within the agreed timelines.
- Oversee the timely reporting of trial results
- Approve / comment on the statistical analysis plan
- Considering new information relevant to the trial e.g. result form other studies that may have a bearing to the conduct of the study and deciding on appropriate action.
- Endorsing annual report to the Funder (if required).

The TSC may recommend early termination of the trial or modification of the study design in the event of a clear outcome derived from accumulating data or on the basis of the information available from other sources or on safety grounds.

The TSC should be available to provide independent advice electronically as required not just when meetings are scheduled.

The TSC should maintain the confidentiality of all information it receives. Members should not discuss confidential issues from their involvement in the study until the primary results have been published.

19.3. Role of the TSC Chair

- With assistance of the PsiDeR CI/PI arranged the TSC meeting to agree the content of the charter and set up schedule of the meetings.
- Establish clear reporting lines – to the Funder, sponsor etc.
- Provide an independent, experienced opinion if conflicts arise between the needs of the research team, the Funder, the Sponsor and/or any other agencies.
- Leading the TSC to provide regular, impartial oversight of the trial, especially to identify and pre-empt problems.
- Ensuring that changes to the protocol are debated and endorsed by other members of the TSC. This may be necessary outside of the planned TSC meetings.

For decisions to be made, at least 2 independent members of the TSC should be present (including the Chair), the CI/PI and representative of TMG.

20. Organisation of meetings

The responsibility for calling and organising TSC/DMC meetings lies with the CI/PI in association with the Chair. The Chair, assisted by the CI/PI, is responsible for facilitating the meetings and summarise discussions. The Funder (King's College London/South London & Maudsley NHS Foundation Trust)

with the help of PI will approve and invite the TSC/DMC membership. The Chair will approve the appointment of the members of the TSC/DMC at the first meeting.

The TSC/DMC membership is for the duration of the trial/study. If members leave the committee, the TMG should provide replacements promptly for appointment by the Chair.

20.1. TSC Meetings

- The responsibility of calling and organising a TSC/DMC meeting lies with the PsiDeR CI/PI in association with the TSC Chair.
- The organising itself may be delegated to the PsiDeR Trial PI or Trial Assistant.
- All TSC/DMC members will be provided with study documents (e.g. protocol, proposed statistical analysis plan (SAP), Patient Information Sheets (PIS), Case Report Forms (CRF), etc, and the TSC report prior to the meeting.
- The first TSC meeting should ideally be held face-to-face to discuss, revise and finalise the terms of reference, agree the content of the TSC charter and TSC report and sign any declaration, and agree the frequency of the meetings.
- The exact timing and /or frequency of subsequent meeting should be no longer than six monthly and usually following each trial statistician report.
- Meetings can also be held at any time at the request of the CI/PI or TSC chair.
- The final TSC meeting will be arranged when target recruitment is completed, all data collected and cleaned, and database is locked. This final meeting will be held to discuss final/completed data and interpretation, and publication timeline. If the trial is terminated prematurely, no final trial meeting is required.

20.2. Attendance

Every effort will be made to ensure that all TSC/DMC members can attend the meetings. The trial manager, PI or delegate (trial assistant) should try and find a date that enables this. The CI/PI must try to attend all meetings, especially if major actions are expected.

67% of the Independent members of the TSC (including the Chair) need to be present for a meeting to take place.

Every effort should be made for all the TSC members to attend. The PsiDeR trial team will try to ensure that a date is chosen to enable this. Members who cannot attend in person will be invited to attend by teleconference. All reasonable expenses for travel will be reimbursed by the PsiDeR trial team.

If a member does not attend a meeting it should be ensured that the member is available for the next meeting. If a member does not attend in the second meeting, they should be asked if they wish to remain part of the TSC. If a member does not attend a third meeting, they should be replaced. If a member cannot attend a meeting but would still like to provide comment, they can do so by documenting their contribution electronically to the TSC Chair for dissemination during the meeting.

If the TSC is considering major actions, the TSC Chair should communicate with absent members, including the CI/PI, as soon after the meeting as possible to determine whether they all agree. If there is disagreement amongst absent members a further meeting should be arranged with the full TSC.

21. Trial documentation and procedures to ensure confidentiality and proper communication

A short report will be prepared by the trial statistician following a standard template. This will report on accrual, recruitment and any matters affecting the trial. No trial outcome measure data will be presented by arm. Where relevant, accrual, compliance with follow-up and adherence to treatment may be presented.

It is usually helpful for the TSC to receive the TSC/DMC report at least 1 week and preferably at least 2 weeks before any meetings.

The accumulating trial data by arm and interim analyses will be confidential. The TSC will not be routinely privy to these interim reports.

Formal statistical methods may be considered to make recommendations to the TSC. These methods are usually used as guidelines rather than absolute rules.

TSC members would be expected to delete, destroy or store securely copies of the reports to and from the TSC, agenda and minutes, as well as copies of communications between meetings. All documentation should be considered confidential. The PI/Facilitator/Trial Assistant will keep a central record of all minutes, reports and correspondence by the TSC.

22. Decision making

Possible decisions include:

- No action needed; trial continues as planned.
- Early stopping due, for example, to clear harm of a treatment.
- Sanctioning and/or proposing protocol changes

There will be no formal interim analyses or stopping rules. We do not plan an interim analysis given that the study is primarily aimed at feasibility.

23. Reporting

Prior to a TSC meeting a report will be prepared by the TMG with input from the trial statistician, CI/PI and trial assistant and circulate to TSC members at least a week (or as otherwise agreed) before the meeting.

On consideration of the information presented at these meetings, the TSC should provide recommendations of appropriate action in writing to the TMG who will be responsible for implementing any actions.

Minutes of the meeting including key points and actions will be prepared by the trial assistant. These minutes will describe the proceedings and include the recommendations of the TSC. All members of the TSC must agree the minutes and this will be signed of by the TSC Chair on behalf of all members. Minutes will be circulated to all TSC members, the TMG, the Sponsor and the trial Funder. Approved Minutes will be filled in the Trial Master File.

Decision and recommendations by the TSC should be unanimous if not a vote may be taken. The role for the Chair is to summarise discussions and encourage consensus. Therefore, it is best for the chair to give their opinion last. It is important that the implications (ethical, statistical, practical and financial) for the trial be considered before any decision is made.

24. Content of the TSC Reports

An outline/Example of the TSC report is given below:

Note – the exact contents to be decided by the TMG and TSC depending on the nature of the trial.

- Outline of the study design, sample size sought and current available evidence
- Statistical consideration and design
- Major protocol amendments
- Patient screening
- Eligibility violations
- Protocol violations by investigators or participants
- Study accrual by month/total
- CRF completed, entry into database
- Baseline characteristics
    - Demographics
    - Disease characteristics
    - Previous treatment usage
    - Laboratory records
- Individual safety reports (executive members only)
- Follow up compliance rate.

25. Conflicts of interest

TSC members should not have any apparent financial, scientific or intellectual conflict of interest that could prevent them from objectively viewing the study protocol, interim and final data and giving advice to the TMG. TSC members should disclose to the chair any other conflicts they consider relevant. Any members who develop significant conflicts of interest during the course of the trial should resign from TSC.

26. Publication

At the end of the trial there may be a meeting to allow the committee to discuss the final data with the key members of TMG and give advice about data interpretation. Manuscripts that arose from the trial will be shared with the TSC and members will be able to comment. The TSC members and their affiliations will be acknowledged in reports of the trial.

(Annexe 1): Agreement and competing interests form for independent members

PsiDeR Trial Steering Committee: Agreement to join the Trial Steering Committee as an independent member and disclosure of potential competing interests.

Please complete the following document and return to the TSC PI/Facilitator.

(please initial box to agree)

☐	I have read and understood the TSC/DMC Charter version 1.0, dated 16/01/2020
☐	I agree to join the Trial Steering Committee for this trial as an independent member
☐	I agree to treat all sensitive trial data and discussions confidentially

The avoidance of any perception that independent members of a TSC may be biased in some fashion is important for the credibility of the decisions made by the TSC and for the integrity of the trial.

Potential competing interests should be disclosed via the Facilitator or PI. In many cases simple disclosure up front should be sufficient. Otherwise, the (potential) independent TSC member should remove the conflict or stop participating in the TSC. **Table 1** lists potential competing interests.

☐	**No,** I have no potential competing interests to declare
☐	**Yes,** I have potential competing interests to declare (please detail below)

Please provide details of any potential competing interests:

____

____

____

Name: ___________________________

Signed: __________________________ Date: ______________

Table 1. Potential competing interests for independent members

- Stock ownership in any commercial companies involved
- Stock transaction in any commercial company involved (if previously holding stock)

- Consulting arrangements with the Sponsor/Funder
- Ongoing advisory role to a company providing drugs to the trial
- Frequent speaking engagements on behalf of the intervention
- Career tied up in a product or technique assessed by trial
- Hands-on participation in the trial
- Involvement in the running of the trial
- Emotional involvement in the trial
- Intellectual conflict e.g. strong prior belief in the trial's experimental arm
- Involvement in regulatory issues relevant to the trial procedures
- Investment (financial or intellectual) or career tied up in competing products
- Involvement in the writing up of the main trial results in the form of authorship

The definition of independent is as follows:

- Not part of the same institution as any of the applicants or members of the project team
- Not part of the same institution that is acting as a recruitment or investigative centre
- Not related to any of the applicants or members of the project team
- For the chair only – not an applicant on a rival proposal

Annexe 2. Agreement and competing interests form for non-independent members

PsiDeR TSC: Agreement to join the Trial Steering Committee as an non-independent member and disclosure of potential competing interests

Please complete the following document and return to the Facilitator.

(please initial box to agree)

☐	I have read and understood the TSC Charter version 1.0, dated 16/01/2020
☐	I agree to join the Trial Steering Committee for this trial as an non-independent ember
☐	I agree to treat all sensitive trial data and discussions confidentially

The avoidance of any perception that members of a TSC may be biased in some undisclosed fashion is important for the credibility of the decisions made by the TSC and for the integrity of the trial.

Possible competing interests should be disclosed via the Facilitator or CI. In many cases simple disclosure up front should be sufficient. Otherwise, the (potential) independent TSC member should remove the conflict or stop participating in the TSC. **Table 2** lists potential competing interests.

☐	**No,** I have no competing interests to declare other than involvement in the trial
☐	**Yes,** I have competing interests to declare (please detail below)

Please provide details of any competing interests:

____

____

____

Name: ____________________________

Signed: ____________________________ Date: _______________

Table 2. Potential competing interests for non-independent members

Stock ownership in any commercial companies involved
Stock transaction in any commercial company involved (if previously holding stock)

General financial conflicts (E.g personal funding)
Consulting arrangements with the Sponsor/Funder
Frequent speaking engagements on behalf of the intervention
Career tied up in a product or technique assessed by trial
Hands-on participation in the trial
Involvement in the running of the trial
Emotional involvement in the trial
Intellectual conflict e.g. strong prior belief in the trial's experimental arm
Involvement in regulatory issues relevant to the trial procedures
Involvement in the writing up of the main trial results in the form of authorship

Note: This definition was derived from the NIHR HTA Research Governance Guidelines for Data Monitoring (and Ethics) Committee – DM(E)C, version date: 25 April 2012

27. References

1. Lingjaerde O, Ahlfors UG, Bech P, et al. The UKU side effect rating scale. A new comprehensive rating scale for psychotropic drugs and a cross-sectional study of side effects in neuroleptic-treated patients. *Acta Psychiatrica Scandinavica Supplementum* 1987;334:1–100.

2. Sheehan DV, Lecrubier Y, Sheehan KH, et al. The Mini-International Neuropsychiatric Interview (M.I.N.I.): the development and validation of a structured diagnostic psychiatric interview for DSM-IV and ICD-10. *Journal of Clinical Psychiatry* 1998;59 Suppl 2:22–57.

3. Hamilton M. A RATING SCALE FOR DEPRESSION. *Journal of neurology, neurosurgery, and psychiatry* 1960;23(1):56–62.

4. Fekadu A, Wooderson S, Donaldson C, et al. A Multidimensional Tool to Quantify Treatment Resistance in Depression. *Journal of Clinical Psychiatry* 2009;70(2):177–84.

5. Montgomery SA, \AAsberg M. A New Depression Scale Designed to be Sensitive to Change. *The British Journal of Psychiatry* 1979;134(4):382–89.

6. Posner K, Brown GK, Stanley B, et al. The Columbia–Suicide Severity Rating Scale: Initial Validity and Internal Consistency Findings From Three Multisite Studies With Adolescents and Adults. *The American journal of psychiatry* 2011;168(12):1266–77.

7. Young RC, Biggs JT, Ziegler VE, et al. A Rating Scale for Mania: Reliability, Validity and Sensitivity. *The British Journal of Psychiatry* 1978;133(5):429–35.

8. McGuire-Snieckus R, McCabe R, Catty J, et al. A new scale to assess the therapeutic relationship in community mental health care: STAR. *Psychological Medicine* 2007;37(1):85–95.

9. Rush JA, Trivedi MH, Ibrahim HM, et al. The 16-Item Quick Inventory of Depressive Symptomatology (QIDS), clinician rating (QIDS-C), and self-report (QIDS-SR): a psychometric evaluation in patients with chronic major depression. *Biological Psychiatry* 2003;54(5):573–83.

10. Spitzer RL, Kroenke K, Williams JBW, et al. A brief measure for assessing generalized anxiety disorder: the GAD-7. *Arch Intern Med* 2006;166(10):1092–97. doi: 10.1001/archinte.166.10.1092

11. Pennebaker JW, Susman JR. Childhood Trauma Questionnaire. Measurement Instrument Database for the Social Sciences. Retrieved from www.midss.ie2013.

12. Spielberger C, Gorsuch R, Lushene R, et al. Manual for the State Trait Anxiety Inventory. Palo Alto: Consulting Psychologists Press 1983.

13. Rosenbaum JF, Fava M, Hoog SL, et al. Selective serotonin reuptake inhibitor discontinuation syndrome: a randomized clinical trial. *Biological Psychiatry* 1998;44(2):77–87.

14. Studerus E, Gamma A, Vollenweider FX. Psychometric Evaluation of the Altered States of Consciousness Rating Scale (OAV). *PLoS One* 2010;5(8):e12412.

15. Altman EG, Hedeker D, Peterson JL, et al. The Altman Self-Rating Mania Scale. *Biological Psychiatry* 1997;42(10):948–55.

16. Derogatis LR, Lipman RS, Covi L. SCL-90: an outpatient psychiatric rating scale–preliminary report. *Psychopharmacological Bulletin* 1973;9(1):13–28.

17. Herdman M, Gudex C, Lloyd A, et al. Development and preliminary testing of the new five-level version of EQ-5D (EQ-5D-5L). *Quality of life research : an international journal of quality of life aspects of treatment, care and rehabilitation* 2011;20(10):1727–36.

18. Mundt JC, Marks IM, Shear MK, et al. The Work and Social Adjustment Scale: a simple measure of impairment in functioning. *Br J Psychiatry* 2002;180:461–4. doi: 10.1192/bjp.180.5.461

19. Tellegen A, Atkinson G. Openness to absorbing and self-altering experiences ("absorption"), a trait related to hypnotic susceptibility. *Journal of abnormal psychology* 1974;83(3):268–77.

20. Watson D, Clark LA, Tellegen A. Development and validation of brief measures of positive and negative affect: the PANAS scales. *Journal of personality and social psychology* 1988;54(6):1063–70.

21. Neff KD. The Development and Validation of a Scale to Measure Self-Compassion. *Self and Identity* 2003;2(3):223–50.

22. Tennant R, Hiller L, Fishwick R, et al. The Warwick-Edinburgh Mental Well-being Scale (WEMWBS): development and UK validation. *Health and quality of life outcomes* 2007;5(1):63.

23. Mayer FS, Frantz CM. The connectedness to nature scale: A measure of individuals' feeling in community with nature. *Journal of Environmental Psychology* 2004;24(4):503–15.

24. Moran P, Leese M, Lee T, et al. Standardised Assessment of Personality – Abbreviated Scale (SAPAS): Preliminary validation of a brief screen for personality disorder. *The British Journal of Psychiatry* 2003;183(3):228–32.

25. Fekadu A, Donocik JG, Cleare AJ. Standardisation framework for the Maudsley staging method for treatment resistance in depression. *BMC Psychiatry* 2018;18(1):100.

26. McIntyre RS, Best MW, Bowie CR, et al. The THINC-Integrated Tool (THINC-it) Screening Assessment for Cognitive Dysfunction. *Journal of Clinical Psychiatry* 2017;78(7):873–81.

27. Bland AR, Roiser JP, Mehta MA, et al. EMOTICOM: A Neuropsychological Test Battery to Evaluate Emotion, Motivation, Impulsivity, and Social Cognition. *Frontiers in Behavioral Neuroscience* 2016;10(339):17–25.

28. Williams JBW, Kobak KA. Development and reliability of a structured interview guide for the Montgomery-Asberg Depression Rating Scale (SIGMA). *The British Journal of Psychiatry* 2008;192(1):52–58.

29. Dittrich A. The standardized psychometric assessment of altered states of consciousness (ASCs) in humans. *Pharmacopsychiatry* 1998;31 Suppl 2(S 2):80–84.

30. Patrick F, Young AH, Williams SC, et al. Prescreening clinical trial volunteers using an online personality questionnaire. *Neuropsychiatric Disease and Treatment* 2018;Volume 14:2297-303. doi: 10.2147/NDT.S169469
